# Brain dysconnectivity patterns associated with chronic back pain development

**DOI:** 10.1186/s40708-026-00328-8

**Published:** 2026-08-21

**Authors:** Stephan Wunderlich, Enrico Schulz, Florian Ringel, Veit M. Stoecklein, Sophia Stoecklein

**Affiliations:** 1 Department of Radiology, LMU University Hospital, LMU Medizin, Ludwig-Maximilians-Universität München, Munich, Germany; 2 Department of Neurosurgery, LMU University Hospital, LMU Medizin, Ludwig-Maximilians-Universität München, Munich, Germany

**Keywords:** FMRI, Chronic Back Pain, Prediction

## Abstract

Chronic back pain often emerges from a transitional period of subacute pain, yet no clinically applicable biomarker exists to identify which patients are at risk for chronification. Evidence suggests that this transition is driven not only by nociceptive input but by changes in brain networks involved in valuation, emotion regulation, and learning. Here, we used resting-state functional magnetic resonance imaging (rs-fMRI) and machine learning to explore whether dysconnectivity in these networks is associated with later development of chronic back pain. We analyzed functional connectivity in 46 patients with subacute back pain and 43 healthy controls from a publicly available longitudinal cohort, classifying patients one year later as either recovered or chronified based on pain outcomes. A data-driven model identified a set of six brain regions whose patterns of dysconnectivity distinguished the two patient trajectories with an area under the curve of 0.87. These regions encompass prefrontal, temporal, and somatosensory hubs implicated in reinforcement learning, avoidance behavior, and pain catastrophizing, suggesting a potential link between dysconnectivity patterns and psychological processes implicated in pain persistence. Based on these features, we introduced an exploratory rs-fMRI–based marker for pain chronification, suggesting potential prognostic relevance that requires independent validation before clinical stratification or targeted intervention can be considered.

## Introduction

While acute back pain serves as an adaptive physiological response to a noxious stimulus [1–3], chronic back pain (CBP) represents a distinct pathological condition. Once pain persists beyond three months, thereby meeting the criteria for CBP, the neuroplastic changes involved, transition from adaptive to maladaptive [4]. In combination with psychological and social factors, this maladaptive reorganization fosters a self-reinforcing cycle that exacerbates and sustains CBP, contributing to its persistence and severity [5–8].

A key question in back pain research is why some patients transition from subacute back pain (SBP) to CBP while others experience recovery (REC). Epidemiological studies suggest that this transition occurs in approximately 9 to 35% of cases [9], leading to substantial costs and a marked reduction in quality of life [10], underscoring the need to identify the factors that drive chronicity. Resting-state functional magnetic resonance imaging (rs-fMRI) has shown potential in predicting this transition, suggesting that SBP may involve a shift from a predominantly sensory-driven representation to one that is increasingly influenced by reinforcement learning and emotional processing [11, 12]. This highlights the pivotal role of affective and emotional mechanisms in the development and persistence of CBP [13–15].

Among the most reliable predictors in rs-fMRI research of the transition from SBP to CBP is the functional connectivity between the medial prefrontal cortex (mPFC) and the nucleus accumbens (NAc), a pathway involved in reward processing. Increased connectivity between these regions has been associated with a higher likelihood of developing CBP, suggesting that dysregulation in the reward system is pivotal in transitioning from SBP to CBP [13, 15, 16]. Moreover, the orbitofrontal cortex (OFC), a major hub in pain modulation [17–19] and reinforcement learning, has been implicated in the persistence of pain, with altered connectivity in CBP patients suggesting negative reinforcement learning amplifies CBP symptoms [12, 20, 21]. Several other neural markers have been implicated as potential predictors for the transition to CBP, reflecting the complex interplay between sensory, affective and cognitive processes. Prior studies have reported altered connectivity in the default mode network (DMN), anterior cingulate cortex (ACC), insula, thalamus, amygdala, hippocampus, and dorsolateral prefrontal cortex (DLPFC), highlighting their roles in pain modulation, emotional regulation, and memory-related reinforcement of pain [13, 15, 22–24].

*Scientific gap.* Most of the CBP studies primarily rely on group-level predictions, limiting their applicability for personalized prognostication, which is crucial, as highlighted by [25, 26]. Individual variability in SBP and CBP connectivity patterns further complicates the identification of universal mechanisms, underscoring the need for a more integrative and personalized approach [26–28]. Despite advances in neuroimaging, clinically validated rs-fMRI biomarkers for predicting CBP transitions remain unavailable, merely differentiating CBP patients from healthy controls [29–31]. This study presents an entirely data-driven approach that leverages rs-fMRI and longitudinal clinical data to identify brain regions critical for transitioning to CBP. To capture patient-specific deviations in functional connectivity, we used dysconnectivity count (DCC), an individualized summary measure of abnormal connectivity relative to a healthy reference distribution. Unlike conventional pairwise functional connectivity analyses, which evaluate the strength of individual connections, DCC quantifies how many connections of a given brain region deviate markedly from the normative range observed in healthy controls. This approach reduces the dimensionality of whole-brain connectivity matrices and emphasizes individualized network abnormalities. We therefore use DCC as a complementary approach to conventional functional connectivity metrics, not as a replacement for them. A key strength of this approach is its ability to account for individual differences in functional connectivity, offering a personalized analysis of each patient’s brain activity relative to a healthy baseline [32–34].

## Materials and methods

A back pain study from the OpenPain database [13, 35], freely available, was utilized. The initial data collection was conducted with the approval of Northwestern University’s Institutional Review Board, ensuring that all participants provided informed consent. Since this study involves a secondary analysis of publicly accessible data, no further ethical approval was necessary. The dataset includes an initial rs-fMRI session conducted while all patients were in the SBP phase. Importantly, spinal MRI examinations were not conducted. Additionally, clinical data were collected, establishing baseline scores for pain and mood using self-report questionnaires, including the McGill Pain Questionnaire Affective (MPQ_A) (Melzack, 1975), McGill Pain Questionnaire Sensory (MPQ_S) [36], McGill Pain Questionnaire Radiculopathy (MPQ_R) [36], McGill Pain Questionnaire Visual Analog Scale (MPQ_VAS) [36–38], the Neuropathic Pain Scale (NPS) [39], the Pain Disability Index (PDI) [40], the Beck Depression Inventory (BDI) [41], and the Positive and Negative Affect Schedule (PANAS) [42]. The study included follow-up clinical and imaging sessions for a maximum of five sessions, with the final session occurring 1074.25 ± 60.56 days after the initial contact. In total, 46 SBP patients (50% female, mean age: 44.13 ± 10.3 years) and 43 healthy controls (46.51% female, mean age: 37.01 ± 7.92 years) were analyzed (Table 1). To categorize patients into REC and CBP groups, we applied criteria from previous research [15]. SBP patients were classified as REC if they exhibited a 20% reduction in MPQ_VAS score over one year, as determined by the Last Session (LS) algorithm. Patients who did not meet this threshold were categorized as CBP patients [15].

**Table 1:** Descriptive characteristics of REC, CBP and the healthy cohort

	REC		CBP		HC	
	(*N* = 23)		(*N* = 23)		(*N* = 43)	
Sex (m : f)	13 : 10		10 : 13		23 : 20	

	Mean	SD	Mean	SD	Mean	SD
Age	41.00	12.07	47.00	7.24	37.01	7.92

Table 1 shows the patients' characteristics with age and sex distribution. Recovery (REC), chronic back pain (CBP),healthy control (HC), standard deviation (SD)

### Image preprocessing

Preprocessing of rs-fMRI was performed using fMRIPrep, Version 23.1.4 [43], based on the *Nipype* toolbox [44]. The initial five functional images were discarded, and the remaining frames underwent standardization and 6.0 mm FWHM Gaussian smoothing. Motion artifacts were corrected using ICA-AROMA and regression of global signal, cerebrospinal fluid, and white matter [45]. The preprocessing was completed with bandpass filtering (0.01–0.08 Hz) and detrending. Brain parcellation was performed using the Automated Anatomical Labeling (AAL) atlas [46]. For each patient, a correlation matrix of the Blood-Oxygen-Level-Dependent (BOLD) signal was computed for the AAL regions in both hemispheres and subsequently normalized using z-scoring. All subjects included in this study met the quality control criteria of temporal signal-to-noise ratio  > 100 and of mean framewise displacement < 0.3 mm [47].

## Dysconnectivity estimation

To measure dysconnectivity patterns, we computed the dysconnectivity count (DCC) per AAL region using the methodology established in previous research [32–34, 48, 49]. For each SBP patient, each pairwise functional connection was compared with the corresponding connection distribution in the healthy control cohort. A connection was classified as dysconnected when it deviated by more than three standard deviations from the healthy control distribution, corresponding to an uncorrected two-tailed p-value of approximately 0.0027 under the assumption of a normal distribution. This threshold was chosen as a conservative criterion to identify pronounced deviations from normative connectivity while reducing the likelihood that minor interindividual variability or noise would be classified as dysconnectivity. The approach was based on the original dysconnectivity framework, which used a more stringent four-standard-deviation threshold; however, given the smaller sample size and the clinical heterogeneity of the present cohort, we used a three-standard-deviation threshold to retain sensitivity to relevant patient-specific deviations while maintaining a conservative definition of abnormal connectivity. The DCC of each AAL region was then defined as the number of dysconnected connections involving that region. Thus, DCC provides an individualized regional measure of how strongly a patient’s connectivity profile deviates from the normative range observed in healthy controls. To assess the robustness of the findings with respect to the choice of dysconnectivity threshold, we performed an additional sensitivity analysis using a more conservative threshold of |z| > 3.5. DCC values were recalculated using this alternative threshold, and the complete downstream machine-learning analysis was repeated using the resulting dysconnectivity estimates.

## Machine learning

A similar machine learning (ML) approach as in [50] was used to identify specific brain regions that may be critical in predicting the transition from SBP to CBP or REC based on the DCC values. In brief, the model included random forest [51], logistic regression [52] with an elastic net approach and support vector machine [53]. These models were implemented in Python v3.7 using Scikit-learn [54]. For the logistic regression model, we used the elastic net approach combining Ridge and LASSO regressions [55]. Model performance was evaluated using repeated random subsampling with 1,000 iterations. In each iteration, the dataset was randomly divided into 80% training and 20% test data. Importantly, feature selection, model training, and feature weighting were performed exclusively within the training data of each split, whereas the held-out test data were used only for performance evaluation. Thus, no information from the test data entered feature selection, model fitting, or feature weighting. Feature-selection stability was quantified as the proportion of the 1000 training iterations in which each brain region ranked among the six most important features.

To further assess model robustness, we performed a sensitivity analysis using a stratified 70/30 train-test split while preserving class balance between CBP and REC patients. In addition, we performed a permutation analysis with 1,000 label permutations. For each permutation, the CBP and REC outcome labels were randomly shuffled and the complete modeling pipeline, including train-test splitting, feature selection within the training data, model fitting, and evaluation on the held-out test data, was repeated. The empirical permutation *p*-value was calculated as the proportion of permuted analyses yielding an AUC equal to or greater than the observed AUC.

Model performance was evaluated primarily using the area under the receiver operating characteristic curve (AUC), together with accuracy, sensitivity, and specificity. Performance metrics were aggregated across the repeated subsampling iterations and are reported as mean ± standard deviation. In addition, empirical 95% intervals for the AUC were obtained from the distribution of AUC values across the 1,000 repeated subsampling iterations.

## Hypo- and hyperdysconnectivity within the most important brain regions

To further analyze patient connectivity patterns, we categorized the DCC into hyper- and hypodysconnective based on their deviations from the healthy cohort. This categorization was applied to the topmost important brain regions identified by the ML algorithm. Hypodysconnectivity was defined as having a DCC lower than a negative threshold value, while hyperdysconnectivity was defined as having a DCC higher than a positive threshold value (threshold |z| > 3).

## Composite score

To provide an interpretable summary of the dysconnectivity pattern identified by the machine-learning analysis, we derived an exploratory composite score incorporating both the magnitude and direction of dysconnectivity. This score was based on the six most consistently informative regions identified. Because the score and its threshold were derived within the same cohort, it should be interpreted as an exploratory in-sample summary measure rather than as an independently validated clinical prediction tool. The DCC values for each key brain region were standardized to z-scores, ensuring consistency and comparability across regions. To account for the relative contribution of each region, feature weights were derived from the machine-learning analysis by averaging feature-importance values across the repeated training iterations. These averaged importance values were used as weights in the composite score. To determine the differentiation between CBP and REC patients, we employed a structured statistical approach. The first step involved a two-sample t-test to compare the mean composite scores of the two groups. Following the confirmation of significant group differences, we determined an optimal threshold to classify patients using Youden’s Index, which identifies the score that maximizes both sensitivity and specificity [56]. To describe the apparent in-sample classification characteristics of this exploratory score, we computed positive predictive value (PPV), negative predictive value (NPV), and positive and negative likelihood ratios. These metrics were calculated in the same cohort used to derive the score and threshold and therefore should not be interpreted as externally validated performance estimates. Additionally, we assessed positive and negative likelihood ratios (LR+ (sensitivity/1-specificity) and LR- (1-sensitivity/specificity), which indicate how much a given test result increases or decreases the probability of belonging to a specific group.

### Statistics

Normality of the DCC distributions was first assessed using the Shapiro–Wilk test. For regions that satisfied normality (*p* > 0.05), an ANCOVA was performed with group (CBP vs. REC) as the main factor and age, sex, and years of education as covariates. Regions that did not meet normality assumptions were rank-transformed, and a rank-based ANCOVA was applied instead. Raw p-values across all tested regions were subsequently corrected for multiple comparisons using false discovery rate (FDR) adjustment (Benjamini–Hochberg procedure). For comparisons of hyperdysconnectivity versus hypodysconnectivity within each group (CBP or REC) and within each brain region, the normality of each distribution was again assessed using the Shapiro–Wilk test. If both the hyper- and hypodysconnectivity distributions were normally distributed, a paired t-test was used; otherwise, a Wilcoxon signed-rank test was employed. As before, raw p-values were corrected for multiple comparisons using FDR.

## Results

### Grouping

The LS algorithm yielded a significant difference in distinguishing REC from CBP (*p*-value 3.17 × 10^–9^).

### Machine learning

**Fig. 1:**
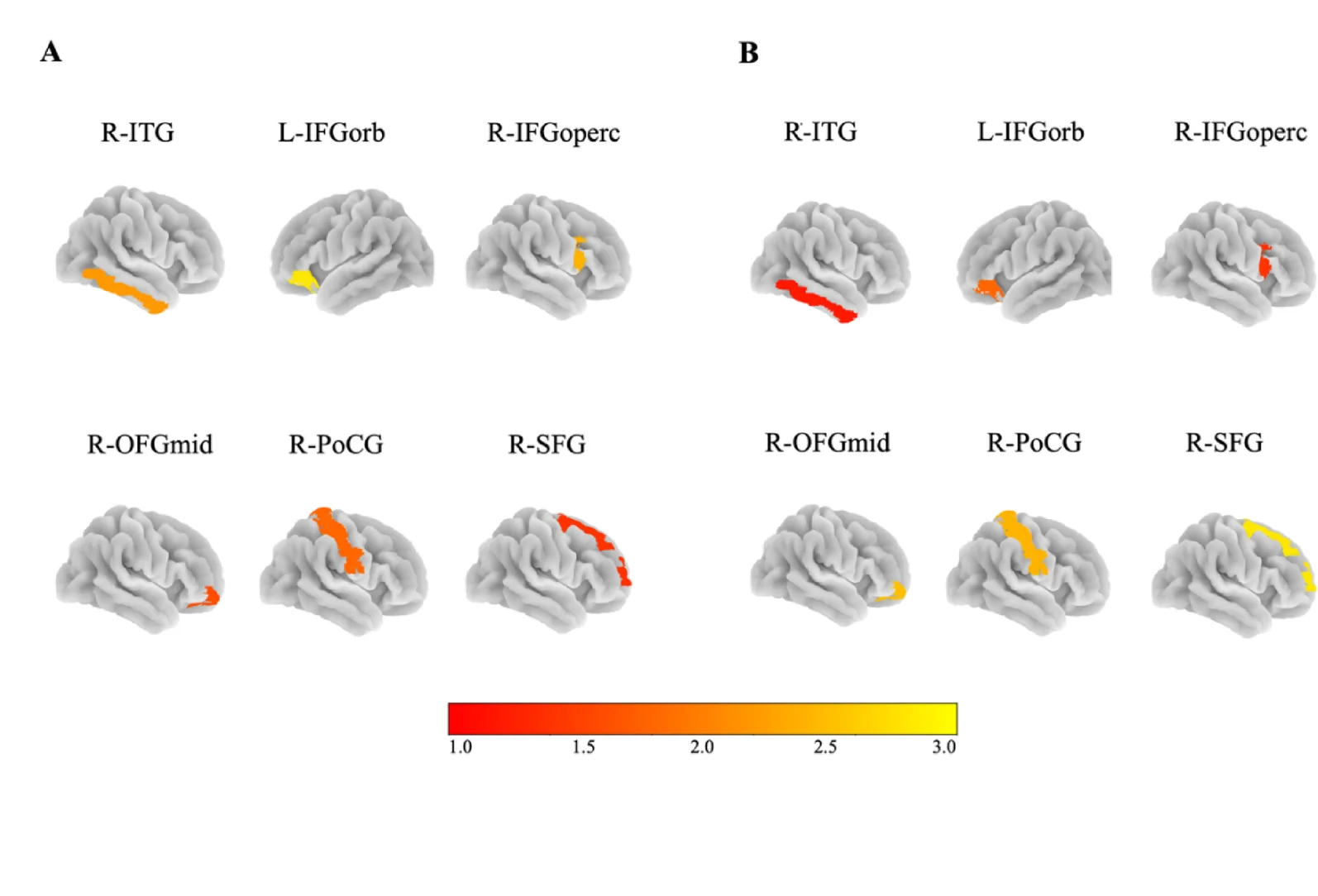
Distribution of dysconnectivity of the most important brain features. Figure 1 illustrates the group-mean distribution of dysconnectivity in chronic back pain and recovery patients. The higher the number (yellow), the more dysconnectivity is present. In panel** A**, CBP patients exhibit pronounced dysconnectivity in R-ITG, L-IFGorb, and R-IFGoperc, with R-OFGmid, R-PoCG, and R-SFG showing moderate levels of dysconnectivity. Conversely, panel** B** shows that in REC patients, higher dysconnectivity is observed in R-OFGmid, R-PoCG, and R-SFG, while R-ITG, L-IFGorb, and R-IFGoperc display lower dysconnectivity. Left inferior frontal gyrus - orbital part (L-IFGorb), right inferior frontal gyrus - opercular part (R-IFGoperc), right inferior temporal gyrus (R-ITG), right orbitofrontal gyrus - medial part (R-OFGmid), right postcentral gyrus (R-PoCG), right superior frontal gyrus (R-SFG)

We observed the highest apparent classification performance using the top six most important features, with a mean AUC of 0.87 ± 0.06, accuracy of 82% ± 9%, sensitivity of 80% ± 8%, and specificity of 84% ± 7% (empirical 95% interval: 0.76–0.97) under repeated subsampling (Table 2; Fig. 1). A permutation analysis using 1000 randomly shuffled outcome-label sets yielded a mean AUC of 0.53 (empirical 95% interval: 0.28–0.72). Only one permutation produced an AUC equal to or greater than the observed value, corresponding to an empirical permutation p-value of 0.002, indicating that the observed classification performance exceeded that expected by chance. Notably, no demographic data were among the topmost important features. Including “age” and “sex” as input for the classification decreased the AUC to 76%. In a more conservative sensitivity analysis using a stratified 70/30 train-test split, model performance decreased to an AUC of 0.81, indicating that the initial estimate may have been optimistic but that the dysconnectivity features retained discriminative capability. Furthermore, using more or less than six features resulted in poorer classification performance. To assess the robustness of the findings to the choice of dysconnectivity threshold, we repeated the complete analysis using a more conservative threshold of |z| > 3.5. Under this alternative threshold, the model achieved an AUC of 0.84, compared with 0.87 using the primary threshold of |z| > 3.0. Importantly, all six of the most important brain regions identified in the primary analysis were retained, indicating that the principal dysconnectivity pattern remained largely unchanged despite the more conservative definition of abnormal connectivity. The most important brain regions identified by the ML algorithm were those that consistently showed high feature importance and were selected most frequently across repeated training iterations. The six regions with the highest average feature importance across splits were the left inferior frontal gyrus, orbital part (L-IFGorb) (frequency selected: 86%), right inferior frontal gyrus, opercular part (R-IFGoperc) (frequency selected: 88%), right inferior temporal gyrus (R-ITG) (frequency selected: 91%), right orbitofrontal gyrus, medial part (R-OFGmid) (frequency selected: 83%), right postcentral gyrus (R-PoCG) (frequency selected: 82%) and right superior frontal gyrus (R-SFG) (frequency selected: 89%). We further examined the individual DCC values of these brain regions in the CBP and REC groups. DCC values for R-ITG, L-IFGorb and R-IFGoperc were significantly higher in the CBP group compared to the REC group (*p* < 0.05 for all). Conversely, the R-OFGmid, R-PoCG and R-SFG (Table 2) showed significantly higher DCC values in the REC group than in the CBP group (*p* < 0.05 for all).

**Table 2:** Statistical analysis of the top 6 most important features

Rank	Weights (ML)	Region	FDR-corrected DCC (*p*-value)	Sex (*p*-value)	Age (*p*-value)	Years of education (*p*-value)
1	0.240	L-IFGorb	*p* < 0.05 (higher in CBP)	*p* = 0.7844	*p* = 0.6933	*p* = 0.7158
2	0.117	R-IFGoperc	*p* < 0.05 (higher in CBP)	*p* = 0.5100	*p* = 0.5294	*p* = 0.3700
3	0.049	R-ITG	*p* < 0.05 (higher in CBP)	*p* = 0.2412	*p* = 0.7955	*p* = 0.8008
4	0.043	R-OFGmid	*p* < 0.05 (higher in REC)	*p* = 0.6870	*p* = 0.7438	*p* = 0.2920
5	0.029	R-PoCG	*p* < 0.05 (higher in REC)	*p* = 0.7927	*p* = 0.6678	*p* = 0.1238
6	0.026	R-SFG	*p* < 0.05 (higher in REC)	*p* = 0.7844	*p* = 0.7931	*p* = 0.1561

Table 2 presents the statistical analysis of the six most important features identified by the machine learning algorithm.Depending on the data distribution, the appropriate statistical test was applied to determine if there were significantdifferences in the DCC values between the recovery and chronic back pain groups. Additionally, sex, age, and yearsof education were included in the analysis to assess their influence, but they did not show a significant effect. The firstthree brain regions exhibit significantly higher DCC values in the CBP group, while the last three brain regions havesignificantly higher DCC values in the REC group. Dysconnectivity count (DCC), recovery (REC), chronic back pain(CBP), left inferior frontal gyrus - orbital part (L-IFGorb), right inferior frontal gyrus - opercular part (R-IFGoperc), right inferior temporal gyrus (R-ITG), right orbitofrontal gyrus - medial part (R-OFGmid), right postcentral gyrus(R-PoCG), right superior frontal gyrus (R-SFG), false-discovery rate (FDR)

### Hyper- and hypodysconnectivity patterns in the topmost important features

**Fig. 2:**
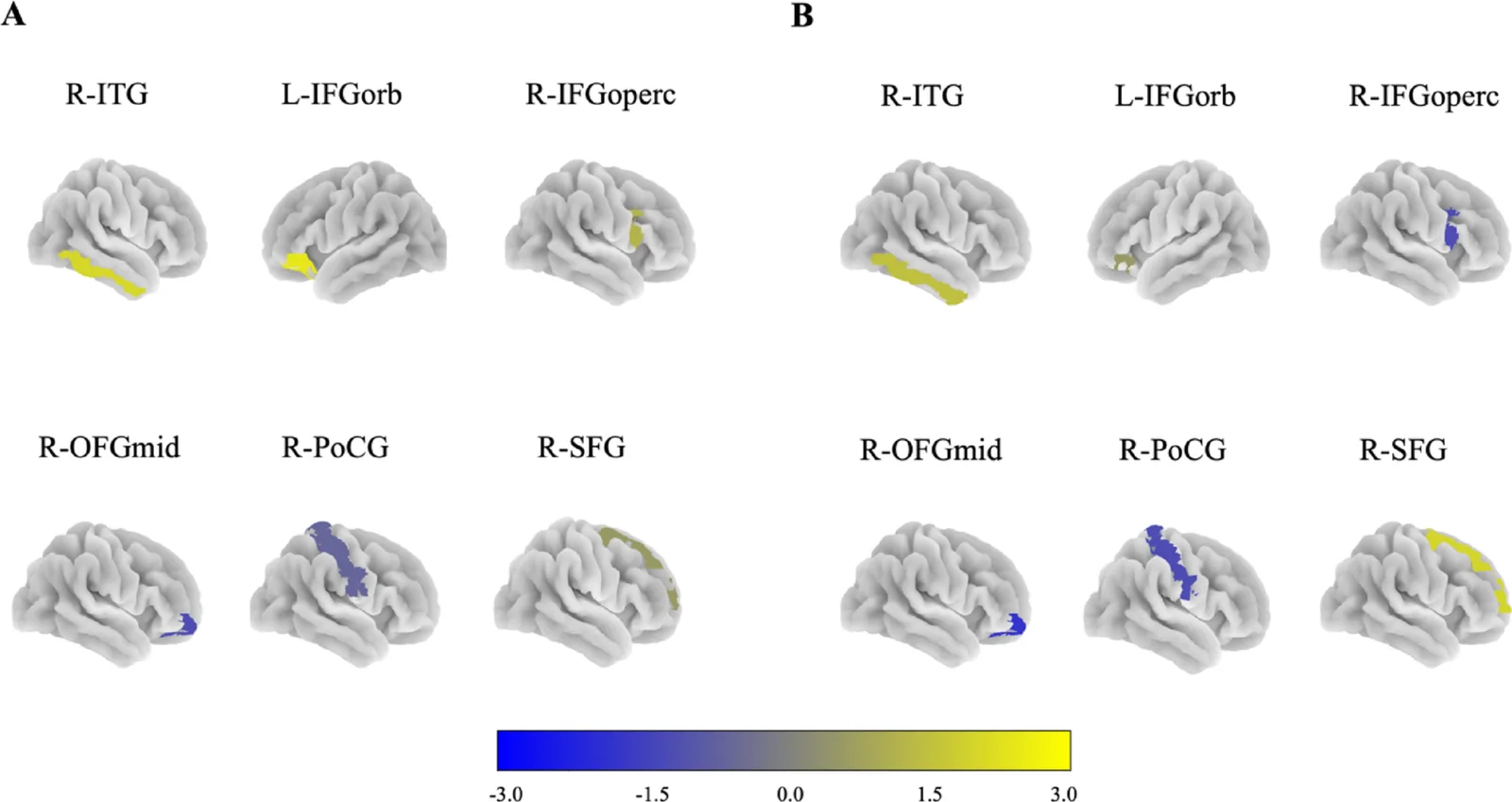
Distribution of Hyper- and Hypodysconnectivity in the Most Important Brain Features. Figure 2 illustrates the group-mean distribution of dysconnectivity in chronic back pain and recovery patients. The higher the number (yellow), the more dysconnectivity is present. In panel** A**, CBP patients exhibit pronounced dysconnectivity in R-ITG, L-IFGorb, and R-IFGoperc, with R-OFGmid, R-PoCG, and R-SFG showing moderate levels of dysconnectivity. Conversely, panel** B** shows that in REC patients, higher dysconnectivity is observed in R-OFGmid, R-PoCG, and R-SFG, while R-ITG, L-IFGorb, and R-IFGoperc display lower dysconnectivity. Left inferior frontal gyrus - orbital part (L-IFGorb), right inferior frontal gyrus - opercular part (R-IFGoperc), right inferior temporal gyrus (R-ITG), right orbitofrontal gyrus - medial part (R-OFGmid), right postcentral gyrus (R-PoCG), right superior frontal gyrus (R-SFG)

We further investigated these six regions by categorizing them into hyper- and hypodysconnected. In the CBP group, dysconnectivity in the R-ITG, L-IFGorb and R-IFGoperc (*p* < 0.05 for all) are significantly driven by hyperdysconnectivity, particularly in the L-IFGorb with the lowest p-value. In the REC group, dysconnectivity is characterized by a mixture of hyper- and hypodysconnectivity, with the R-OFGmid showing significant hypodysconnectivity (*p* < 0.05) (Table 3; Fig. 2).

**Table 3:** Statistical analysis of hyper- and hypodysconnectivity of the most important brain regions

Brain region	FDR-corrected *p*-values	Dysconnectivity driven by hyper- or hypodysconnectivity	
*A. Chronic Back Pain Group*			
R-ITG	*p* < 0.05	Hyperdysconnectivity	
L-IFGorb	*p* < 0.05	Hyperdysconnectivity	
R-IFGoperc	*p* < 0.05	Hyperdysconnectivity	
R-OFGmid	*p* > 0.05	Not significant	
R-PoCG	*p* > 0.05	Not significant	
R-SFG	*p* > 0.05	Not significant	
*B. Recovery Group*			
R-ITG	*p* < 0.05	Hyperdysconnectivity	
L-IFGorb	*p* > 0.05	Not significant	
R-IFGoperc	*p* < 0.05	Hypodysconnectivity	
R-OFGmid	*p* < 0.05	Hypodysconnectivity	
R-PoCG	*p* > 0.05	Not significant	
R-SFG	*p* < 0.05	Hyperdysconnectivity	

Table 3 presents the statistical analysis of the absolute DCC values, categorized into hyper- and hypodysconnectivity for: (A) CBP and (B) REC. In the CBP group, dysconnectivity is primarily driven by hyperdysconnectivity in the first three brain regions. In contrast, the REC group exhibits a mix of hyper- and hypodysconnectivity, with R-OFGmid and R-IFGoperc showing significant hypodysconnectivity, while R-SFG and R-ITG are characterized by hyperdysconnectivity. Regions with significantly greater dysconnectivity in each group relative to the other group are highlighted in yellow (Table 2). Recovery (REC), chronic back pain (CBP), dysconnectivity count (DCC), left inferior frontal gyrus - orbital part (L-IFGorb), right inferior frontal gyrus - opercular part (R-IFGoperc), right inferior temporal gyrus (R-ITG), right orbitofrontal gyrus - medial part (R-OFGmid), right postcentral gyrus (R-PoCG), right superior frontal gyrus (R-SFG), false-discovery rate (FDR)

### Composite-score

The composite score is defined as follows:

$$ \begin{aligned} {\text{Composite Score }} = & {\mathrm{w}}_{1} {\text{ }} \times {\text{ R - ITG }} + {\text{ w}}_{2} {\text{ }} \\ & \times {\text{ L - IFGorb}} + {\text{ w}}_{3} {\text{ }} \times {\text{ R }} \\ & {\text{ - IFGoperc}} - {\text{ w}}_{4} {\text{ }} \times {\text{ R }} \\ & {\text{ - OFGmid}} - {\text{ w}}_{{\mathrm{5}}} {\text{ }} \times {\text{ R }} \\ & {\text{ - PoCG }} - {\text{ w}}_{{\mathrm{6}}} {\text{ }} \times {\text{ R - SFG}} \\ \end{aligned} $$

Here, w represents the weights from the ML algorithm for each region. Positive terms in the formula reflect regions with greater dysconnectivity in CBP, while negative terms reflect regions with greater dysconnectivity in REC. This approach allows the score to summarize both the magnitude and directional pattern of dysconnectivity associated with REC versus CBP in the present cohort. The analysis yielded a mean composite score of 0.441 (SD = 0.303) for the REC group and 1.253 (SD = 0.367) for the CBP group, with a highly significant difference (p-value = 2.15 × $\:{10}^{-10}$). Because the composite score and threshold were derived within the same cohort, the following classification metrics represent apparent in-sample estimates. The optimal threshold for distinguishing between groups was 0.603, based on Youden’s Index. The apparent in-sample PPV was 82.1%, indicating that when a patient is classified as CBP, there is an 82.1% probability that the classification is correct. The apparent in-sample NPV was 81%, meaning that most of the patients classified as REC were correctly identified. The apparent in-sample LR⁺ was 5.0, suggesting that patients with composite scores above 0.603 are 5 times more likely to belong to the CBP group than REC. The apparent in-sample LR⁻ was 0.24, indicating that a composite score below 0.603 provides evidence that the patient will not transition to CBP.

## Discussion

Based on individualized dysconnectivity patterns, we identified six key regions R-ITG, L-IFGorb, R-IFGoperc, R-OFGmid, R-PoCG, and R-SFG, that showed apparent internal predictive relevance in the ML model and contributed to the exploratory composite score distinguishing CBP from REC during the SBP phase. The relatively high AUC observed in the initial analysis should be interpreted with caution. As demonstrated by the sensitivity analysis using a stratified 70/30 split, model performance decreased, highlighting the susceptibility of performance estimates to optimistic bias in small samples. The six identified regions have previously been implicated in processes relevant to pain chronification, including reinforcement learning, avoidance behavior, and pain-related catastrophizing [57–59]. Although the identified regions have previously been implicated in reinforcement learning, avoidance behavior, and pain-related catastrophizing, these psychological constructs were not directly examined in the present study. Consequently, the mechanistic interpretation of the identified dysconnectivity patterns is based on prior literature and should be regarded as hypothesis-generating rather than as direct evidence for these processes.

### Important brain regions with increased dysconnectivity in the CBP group

The IFGorb, identified as the most important feature in the ML model, is closely related to the ventro-mPFC (vmPFC) and functionally part of the OFC [60]. Along with the IFGoperc, it has been implicated in emotion regulation [61, 62], reward processing, reinforcement learning and decision-making [63]. Consistent with existing literature, OFC dysfunction has been associated with CBP, mainly through alterations in reward and motivation circuits [64], which can reinforce maladaptive pain-related behaviors [20, 65–72]. The IFG is associated with cognitive control and inhibitory regulation, suppressing negative thoughts, emotional distress, and avoidance behavior [73, 74]. Dysfunction in this region may contribute to rumination and catastrophizing by impairing the ability to regulate intrusive, pain-related thoughts [13]. Both the IFG and OFC are related with the outcome evaluation and risk assessment and are associated with negative bias, excessive worry, and maladaptive decision-making. These cognitive tendencies may influence pain-related decision-making [66, 75] and contribute to pain catastrophizing.

### Important brain regions with increased dysconnectivity in the REC group

The OFGmid within the vmPFC, a key component of the DMN, plays an important role in decision-making and reward valuation, sharing similar functions as the IFGorb [70, 71]. The different dysconnectivity patterns of OFGmid (higher dysconnectivity in the REC group) and IFGorb (higher dysconnectivity in the CBP group) suggest distinct contributions to pain chronification versus recovery in SBP patients. Besides the overall function of the OFC, where OFGmid and IFGorb are functionally part of, their function differs. Dysconnectivity in the OFGmid, particularly in the context of limbic system interaction (amygdala, hippocampus, and ACC) [76] and social cognition, might facilitate emotional modulation and reward-based evaluation of pain. Within its interaction with the limbic system, avoidance behavior may be modulated, potentially influencing the development of CBP. In contrast, the IFGoperc (higher dysconnectivity in the CBP group) within the IFG might contribute to the cognitive control of pain perception, potentially influencing the development of CBP through its role in regulating pain-related behaviors [77]. Thus, the altered connectivity of OFGmid in REC patients may facilitate emotional resilience. In contrast, greater dysconnectivity in IFGorb in CBP patients may contribute to heightened pain perception and difficulty filtering pain-related signals.

The SFG, a key component of the DMN, showed increased dysconnectivity in REC patients, which may suggest altered cognitive and self-referential processing. PoCG, essential for sensory integration, typically contributes to heightened pain perception and avoidance learning in chronic pain [21, 78]. Its dysconnectivity in REC may reduce pain salience, weakening the link between nociceptive input, avoidance behavior, and catastrophizing by limiting excessive sensory focus [79]. Similarly, SFG, involved in attention and self-referential thinking [80–82], might reinforce pain-related rumination and maladaptive reinforcement learning in CBP. Its dysconnectivity in REC patients may prevent excessive cognitive fixation on pain, reducing catastrophizing and avoidance tendencies.

### The role of hyperdysconnectivity and hypodysconnectivity in CBP and REC

Hyperdysconnectivity, within the IFGorb and IFGoperc regions in the CBP group, may indicate an exaggerated activation of emotion and reward processing networks. It might also lead to heightened emotional responses to pain and higher expectations regarding rewards and reward learning, contributing to CBP [83]. Previous studies have shown that increased functional connectivity between medial prefrontal regions and the NAc is associated with the progression from subacute to CBP. We did not directly replicate this specific connectivity pattern, as the NAc did not emerge among the most important dysconnectivity features. However, we identified prefrontal regions, including the R-OFGmid, which is functionally related to ventromedial prefrontal circuits involved in reward processing. These findings may therefore reflect complementary alterations within prefrontal networks rather than the specific cortico-striatal pathway previously described [13]. Alternatively, developing CBP itself may drive heightened activity in IFGorb and IFGoperc related regions [84]. Furthermore, the hyperdysconnectivity in the CBP patients in our results within the ITG is in line with increased connectivity between the ITG and pain-related regions, which has been linked to heightened pain perception and CBP [85, 86]. In contrast, hypodysconnectivity observed in OFGmid in the REC may facilitate better emotional regulation, less negative reinforcement learning, and cognitive control, aiding pain recovery. Pronounced hypodysconnectivity in the OFGmid observed in REC may reflect normalizing processes of emotional regulation, preventing the transition to CBP.

### Lateralization differences in pain processing

Five of the six most important regions identified in the present study were located in the right hemisphere. Although previous studies have suggested greater right-hemispheric involvement in affective aspects of pain processing, the present study was not designed to investigate hemispheric specialization. Therefore, this observation should be regarded as exploratory and requires confirmation in independent cohorts [86–88].

### Beyond the six-region model for a comprehensive understanding of CBP development

However, while these six regions may be related to processes such as reinforcement learning, avoidance behavior, and catastrophizing based on prior literature, they do not fully account for other known mechanisms in CBP development. Specifically, sensory disinhibition and central sensitization, processes in enhanced pain perception and allodynia, are primarily driven by the thalamus, insula, and periaqueductal gray (PAG) [89–93]. Notably, the AAL Atlas does not contain the PAG. Likewise, emotional dysregulation and affective amplification, which contribute to pain-related distress and heightened pain salience, involved with the amygdala, ACC and hippocampus, are also not part of the top most important features [23, 94, 95]. Additionally, cognitive dysfunction and impaired top-down pain regulation, which make it harder for CBP patients to disengage from pain-related thoughts, are strongly influenced by the DLPFC [24], a region not picked by our model. Furthermore, the habit formation and automatization of CBP are predominantly mediated by the striatum and NAc, which are not represented in the top most important features [13, 96].

### Prognostic value of the analysis and clinical impact

The present findings suggest that dysconnectivity-based models may have prognostic potential for identifying SBP patients at increased risk of developing CBP. The exploratory composite score provides an interpretable summary of the dysconnectivity pattern associated with CBP versus REC in the present cohort. However, because the score, threshold, and classification indices were derived from the same cohort, its clinical utility remains to be established in independent validation datasets.

Furthermore, one might argue that ordering a brain rs-fMRI at the subacute stage could heighten patients’ attention to pain. However, a large-scale MRI cohort study identified depression and anxiety as the strongest predictors of back pain, while spinal imaging findings showed only limited relevance [97]. Conducting rs-fMRI at this earlier phase, when structural spinal changes may not be the primary drivers, can help patients see that subtle alterations in brain connectivity already place them at risk of progressing to chronic pain. This, in turn, may reduce excessive concern about “damage” in the back and encourage engagement in treatments addressing the neurobiological and psychological mechanisms underlying pain. Finally, rs-fMRI may represent a valuable tool for identifying patients at risk of developing CBP early in the subacute phase. These individuals could then be targeted with tailored therapeutic interventions aimed at preventing chronification [98, 99].

### Limitations

This study has several limitations. First, the functional interpretation of the six identified regions is based on prior literature and was not directly tested in the present dataset. Behavioral measures of catastrophizing, avoidance, or reinforcement learning were not analyzed in relation to dysconnectivity patterns; therefore, these mechanistic interpretations remain hypothesis-generating. Moreover, the identified regions do not represent the full network of brain areas involved in CBP development, and the absence of regions such as the thalamus, insula, ACC, DLPFC, striatum, or NAc among the top features should not be interpreted as evidence that these regions are unimportant.

Second, the relatively small sample size (23 CBP and 23 REC patients) limits generalizability and increases the risk of variability, statistical noise, and optimistic bias in the machine-learning estimates. Although feature selection was restricted to the training data within each split, internal validation may still overestimate performance, as also suggested by the reduced AUC in the stratified 70/30 sensitivity analysis. Likewise, because the 1,000 repeated subsampling iterations drew from the same 46 patients rather than from independent samples, the resulting feature-selection frequencies (82–91%) reflect stability within this overlapping resampling scheme and should not be interpreted as evidence that the same regions would be selected with comparable consistency in an independent cohort. Similarly, the composite score, its weights, and its optimal threshold were derived and evaluated within the same cohort; thus, PPV, NPV, and likelihood ratios represent apparent in-sample estimates and require independent validation.

Third, spinal MRI data were unavailable, limiting assessment of structural spinal contributions to pain progression. Finally, rs-fMRI–based connectivity measures cannot determine whether observed hyper- or hypodysconnectivity reflects excitatory or inhibitory neuronal activity. Future studies using larger independent cohorts, alternative dysconnectivity thresholds, behavioral assessments, spinal imaging, and higher-resolution approaches such as 7T MRI may help validate and refine these findings before clinical application can be considered.

## Conclusions

This study introduces an exploratory rs-fMRI–based dysconnectivity marker for back pain progression. Based on a single rs-fMRI session in the SBP stage, the proposed analysis may help identify dysconnectivity patterns associated with later recovery or progression to CBP. This is achieved by applying a machine-learning approach to patient rs-fMRI data and evaluating dysconnectivity in the brain regions that showed the highest predictive relevance in the present cohort. Moreover, this framework links specific dysconnective brain regions to psychological processes implicated in the biopsychosocial model of chronic back pain, including reinforcement learning, avoidance behavior, and catastrophizing. However, these mechanisms were inferred from prior literature and were not directly tested in the present study. Therefore, this interpretation should be considered hypothesis-generating. Identifying patients at risk of transitioning to CBP at an early stage could eventually inform more targeted prevention strategies.

## Data Availability

All data and code used in this study are publicly available via the OpenPain repository: https://openpain.org. The dataset includes neuroimaging, behavioral, and clinical information from the SBP and CBP cohorts used in the current analysis.

## Code availability

Code is available on request.
